# Urolithin A and B Alter Cellular Metabolism and Induce Metabolites Associated with Apoptosis in Leukemic Cells

**DOI:** 10.3390/ijms22115465

**Published:** 2021-05-22

**Authors:** Abdulaziz Musa Alzahrani, Mohammed Razeeth Shait Mohammed, Raed Ahmed Alghamdi, Abrar Ahmad, Mazin A. Zamzami, Hani Choudhry, Mohammad Imran Khan

**Affiliations:** 1 Biochemistry Department, Faculty of Science, King Abdulaziz University, Jeddah 21589, Saudi Arabia; abdalazizbioc@gmail.com (A.M.A.); razeeth.new@gmail.com (M.R.S.M.); raed.gh1993@gmail.com (R.A.A.); abrar.ahmadg@yahoo.com (A.A.); mzamzami@kau.edu.sa (M.A.Z.); hchoudhry@kau.edu.sa (H.C.); 2 Centre of Artificial Intelligence in Precision Medicines, King Abdulaziz University, Jeddah 21589, Saudi Arabia

**Keywords:** urolithin, leukemia, ellagic acid, glutamine, one-carbon metabolism

## Abstract

Leukemia is persistently a significant cause of illness and mortality worldwide. Urolithins, metabolites of ellagic acid and ellagitannins produced by gut microbiota, showed better bioactive compounds liable for the health benefits exerted by ellagic acid and ellagitannins containing pomegranate and walnuts. Here, we assessed the potential antileukemic activities of both urolithin A and urolithin B. Results showed that both urolithin A and B significantly inhibited the proliferation of leukemic cell lines Jurkat and K562, among which urolithin A showed the more prominent antiproliferative capability. Further, urolithin treatment alters leukemic cell metabolism, as evidenced by increased metabolic rate and notable changes in glutamine metabolism, one-carbon metabolism, and lipid metabolism. Next, we evidenced that both urolithins equally promoted apoptosis in leukemic cell lines. Based on these observations, we concluded that both urolithin A and B alter leukemic cell metabolome, resulting in a halt of proliferation, followed by apoptosis. The data can be used for designing new combinational therapies to eradicate leukemic cells.

## 1. Introduction

Despite increased early diagnosis and progress in the therapies, leukemia is still a leading cause of illness and mortality worldwide [1]. Human leukemia is recognized by the frequency of chronic chromosomal translocations, which resulted in the cohort of aberrant oncogenic activities. Possibilities for treatment continue to be limited and are beset with harmful side effects [2,3,4,5]. Successful therapeutic effects of the small molecular inhibitor imatinib has turned it into a model of targeted therapy [6]. In acute promyelocytic leukemia (APL), in connection with which targeted treatment is being designed, transforming it from a very deadly disease to a controllable condition [7], all leukemia patients still receive chemotherapy, which developed more than half a century ago and has a long-term complete reduction only in less than 40% of young patients. The treatment is usually too toxic to be used in old, aged patients above 60 years old [8].

Recent studies, using harmless dietary compounds and many natural compounds, are currently investigating their potential chemopreventive in several clinical trials [9,10,11]. The fruit *Punica granatum* has shown great promise as an anticancer compound in the lung [12], colon [13], skin [14], and breast cancer [15], and it has been undertaken in phase II clinical trials in prostate cancer. Previous studies have shown that crude extract from pomegranate juice induces apoptosis and inhibits the cell cycle in several leukemic cell lines [16]. Active compounds were identified in the polyphenol composition within fractions of pomegranate juice with pure compounds during investigations on potential agents inducing apoptosis and cell cycle arrest. Specifically, urolithin A inhibits cell viability and induces apoptosis in prostate cancer [17], and urolithin B has been shown to reduce cell proliferation via inhibiting Wnt/β-catenin signaling in colon cancer [18].

Urolithins are microbial metabolites produced in the human gut microbiota after consuming ellagitannin-rich foods such as pomegranates and walnuts. There are diverse types of urolithins that have been identified, such as urolithin A, B, C, and D. Previous studies have shown urolithins have antioxidant capacities, as well as anti-inflammatory, estrogenic, and antiestrogenic activities. Urolithins and their derivatives have effective in vitro chemopreventive and chemotherapeutic [19,20,21,22], but several molecular mechanisms, cellular and metabolic mechanisms remain to be determined. Cagaita (*Eugenia dysenterica* DC) fruit containing ellagitannins is metabolized to urolithin and has been shown to reduce glycolytic index in patients [23]. To gain new insights into the role of urolithins in cancer therapy, we investigated the metabolic and antiproliferative effects of the most abundantly produced urolithins in humans, urolithins A and B, in different leukemia cell lines. We aimed to establish metabolic profiling, which may constitute valuable information in understanding urolithins’ mechanism during treatment.

## 2. Results

### 2.1. Urolithin A and B Treatments Inhibit Leukemic Cell Proliferation

**Figure 1:**
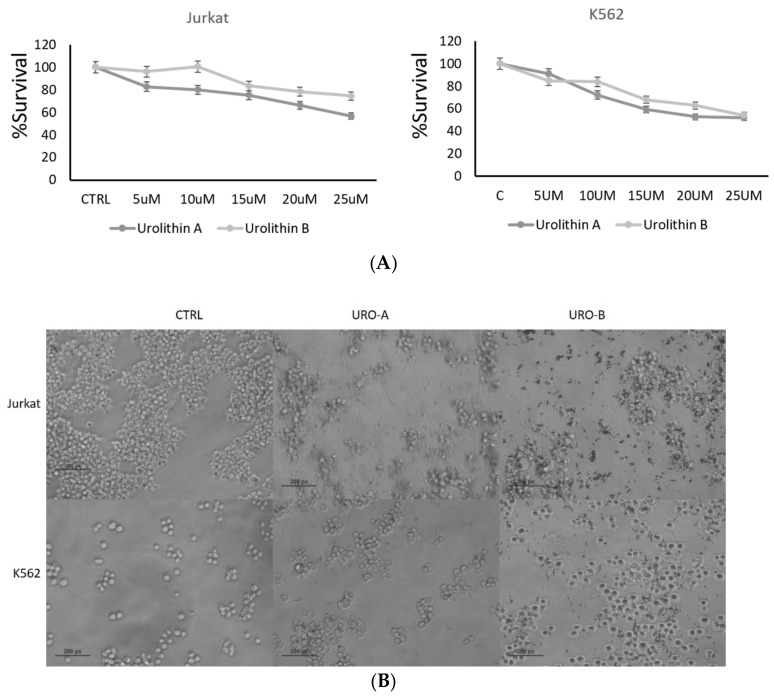
Urolithin A and B treatments reduce cell proliferation and alter cellular morphology (**A**,**B**). MTT assays were performed to determine cell viability upon treating with different concentrations of urolithin A and B for 48 h in both Jurkat and K562. All the images are captured by using a Nikon phase contrast microscope at 20×. *p* < 0.01.

Leukemic cell lines Jurkat and K562 were tested for sensitivity to urolithin A and B in vitro using a cell proliferation assay (Figure 1A). IC50 values of urolithin A and B for these cell lines were determined 48 h after treatment. Briefly, urolithin A and B 25 μmol/mL showed the most sensitive response growth-inhibitory effect in Jurkat and K562. Microscopic examination also showed an increase in the number of cells in apoptotic morphology (chromatin condensation) and decreased the number of cells, showing normal nuclear morphology in treated cells (Figure 1B).

### 2.2. Urolithin A and B Alter the Global Metabolic Landscape of Leukemic Cells

**Figure 2:**
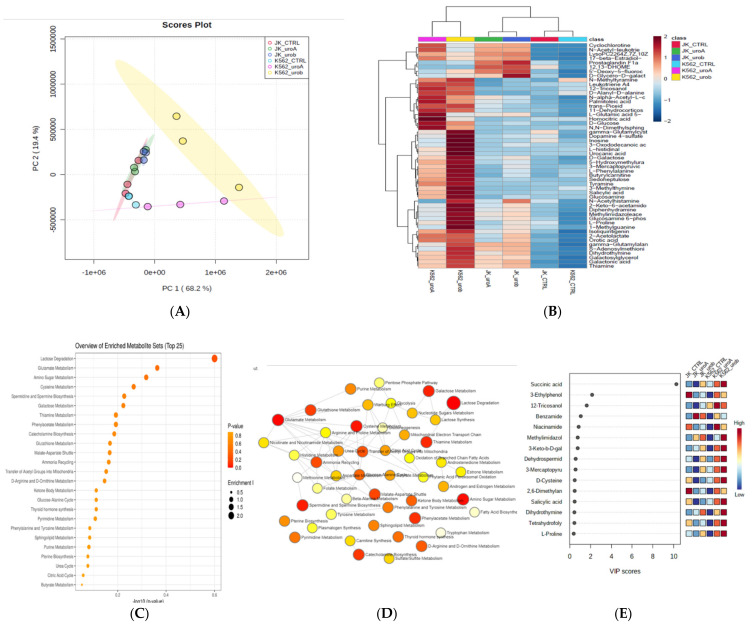
Metabolomic analysis of leukemic cells treated with urolithin A and B for 48 h: (**A**) PCA analysis of total metabolites of Jurkat and K562; (**B**) expression heat map of differential metabolites expressed in control and urolithin A- and urolithin B-treated cells; (**C**) top pathways enriched in Jurkat and K562 control and urolithin A- and urolithin B-treated cells; (**D**) pathway network analysis of Jurkat and K562 control and urolithin A- and urolithin B-treated cells; (**E**) VIP score for differentially expressed metabolites during urolithin A and B treatment.

To understand metabolic alteration of leukemic cells treated with urolithin A and B, metabolites were extracted from urolithin A- and urolithin B-treated Jurkat and K652 cells and examined in LC–MS/MS. The intracellular metabolites were compared between treated and untreated leukemic cells. High-grade spectra of three replicates from the cell lines (Jurkat and K562) were obtained. LC–MS/MS spectral separation of the intracellular metabolic extracts is shown in Supplementary Figure S1, and a comprehensive metabolite list with peak intensity and identification is shown in Table S1. HMDB databases have identified the spectral metabolites. Metabolomic profiles showed variation between samples by PLS–DA score plot (multivariate analysis) (Figure 2A,B); PLS–DA (FDR correction *q* < 0.05 and *p* < 0.05). A metabolites heat map with an FDR-corrected q-value <0.05 and demonstrated by the Ward clustering (Figure 2B) showed the differential metabolite accumulation between control and urolithin treatment. For the pathway enrichment analysis, the differential structured metabolites from the data set have been mapped to the KEGG database using MetaboAnalyst 5.0. A top 25 enriched pathways (Figure 2C–E), significantly with *p* < 0.05 (Supplementary Table S2). The enriched pathways involved in carbohydrate metabolism lactose degradation, glutamate metabolism, amino sugar metabolism, cysteine metabolism, glutathione metabolism, TCA cycle, and sphingolipid metabolism.

### 2.3. Urolithin A and B Alter Levels of Cellular Energy Metabolites

**Figure 3:**
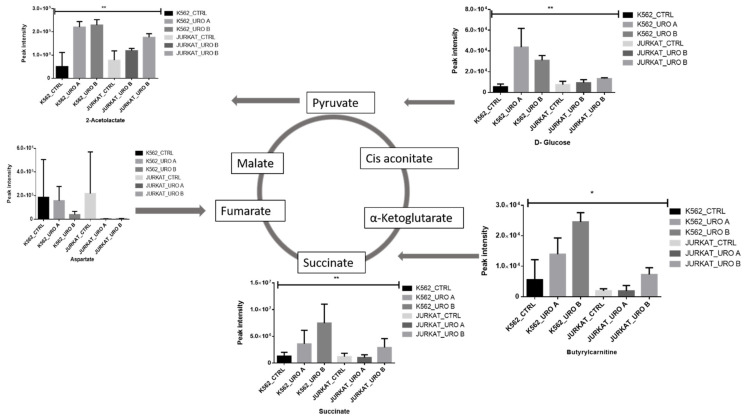
Urolithin A and B both alter crucial cellular energy pathways in leukemic cells. Quantitative levels of various metabolites involved in various energy pathways of control and urolithin A- and urolithin B-treated cells, * *p* < 0.01. ** *p* < 0.001.

To determine the urolithin A and B effects on energy metabolism, the intermediate metabolites involved in glycolysis and the TCA cycle were evaluated. It showed intermediate metabolite, especially glucose, succinic acid, and acetolactate, increased in urolithin A- and urolithin B-treated leukemic cells; this suggests that leukemic cells have switched to more oxidative phosphorylation (OXPHOS) phenotype and regulated by urolithin A and B treatment. (Figure 3). Butryl-carnitine from fatty acid oxidation fuels the TCA cycle through the conversion of succinate. Urolithin treatment induces carnitine levels in treated cells and turns cells toward OXPHOS.

### 2.4. Urolithin A and B Treatments Modify Glutamine Metabolism

**Figure 4:**
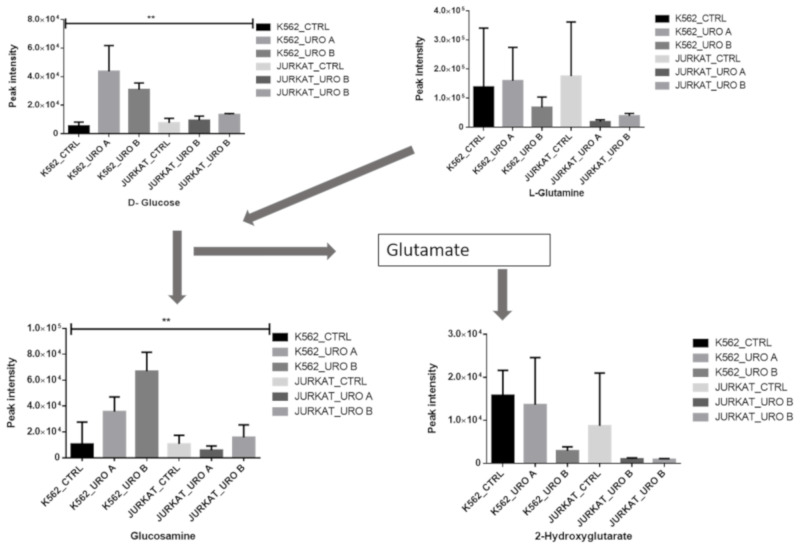
Urolithin A and B both alter glutamine pathways in leukemic cells. Quantitative levels of various metabolites involved in glutamine pathway of control and urolithin A- and urolithin-B treated cells, ** *p* < 0.001.

Glutamine metabolism is essential for maintaining the cellular antioxidative protection mechanism. Glutamate–glutamine is a contribution to glutathione biosynthesis [24]. The urolithin treatment was shown to reduce glutamine and 2-hydroxglutatrate levels. This metabolism has a crucial role in the repression of oxidative stress mechanisms (Figure 4). Glucosamine (GlcN) is salvaged into the hexosamine pathway [25]. It suppresses T cell function by increasing the apoptosis of activated T cells [26]; urolithin treatment induces glucosamine accumulation in leukemic cells.

### 2.5. Urolithin A and B Treatments Modulate One-Carbon Metabolism in the Leukemic Cell Lines

**Figure 5:**
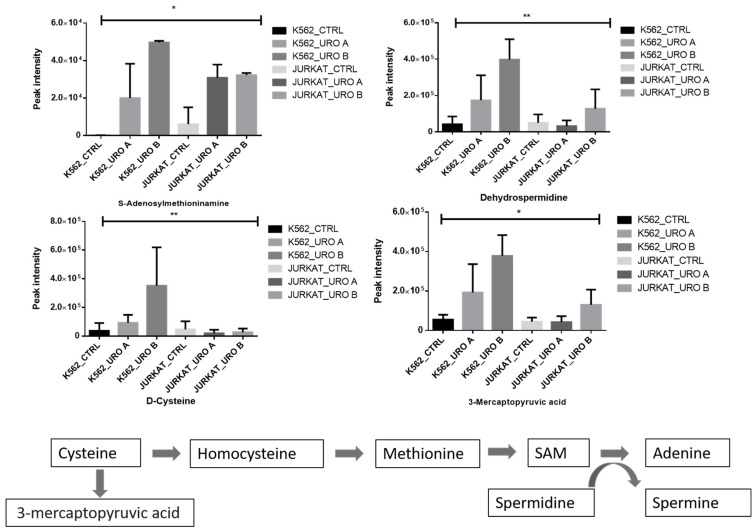
Urolithin A and B both modify one-carbon metabolic pathway in leukemic cells. Quantitative levels of various metabolites associated with one-carbon metabolism pathway of control and Urolithin A- and urolithin B-treated cells. * *p* < 0.01. ** *p* < 0.001.

Methionine is crucial in one-carbon metabolism since methionine is sequentially converted into S-adenosylmethionine (SAM) and will use it in DNA, RNA, and histones methylation. Methionine is derivative of cysteine and a key metabolite involved in one-carbon metabolism. Spermidine involves in the conversation of SAM into adenine. We observed that urolithin treatment showed an increase in SAM and spermine levels (Figure 5). This result shows that urolithin treatment alters the methylation pattern in leukemic cells.

### 2.6. Urolithin A and B Treatments Induce a Level of Fatty Acid Responsible for Cell Death

**Figure 6:**
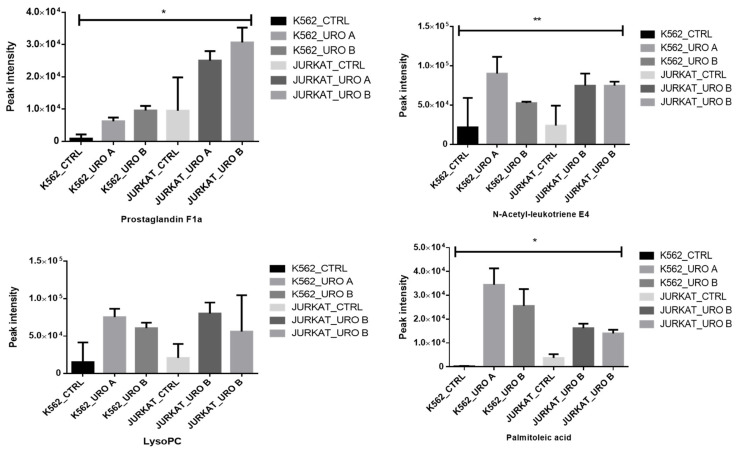
Urolithin A and B both alter various metabolites associated with different lipid pathways in leukemic cells. Data presented here for both control and urolithin A- and urolithin B-treated cells, * *p* < 0.01. ** *p* < 0.001.

Fatty acids regulate the induction of apoptosis in several cell types [27,28]. Short-chain fatty acids reduce histone deacetylases, which resulted in a hyperacetylation of histone proteins [29]. Histone hyperacetylation is related to growth inhibition and transcriptional regulation in colonic epithelial cells. We observed that urolithin treatment showed an increased level of fatty acids LysoPC, prostaglandin F1a, and palmitoleic in leukemic cells, and these fatty acids are a marker for apoptosis (Figure 6).

### 2.7. Urolithin A and B Treatments Increase the Level of DNA Damage-Associated Metabolites in Leukemic Cell Lines

**Figure 7:**
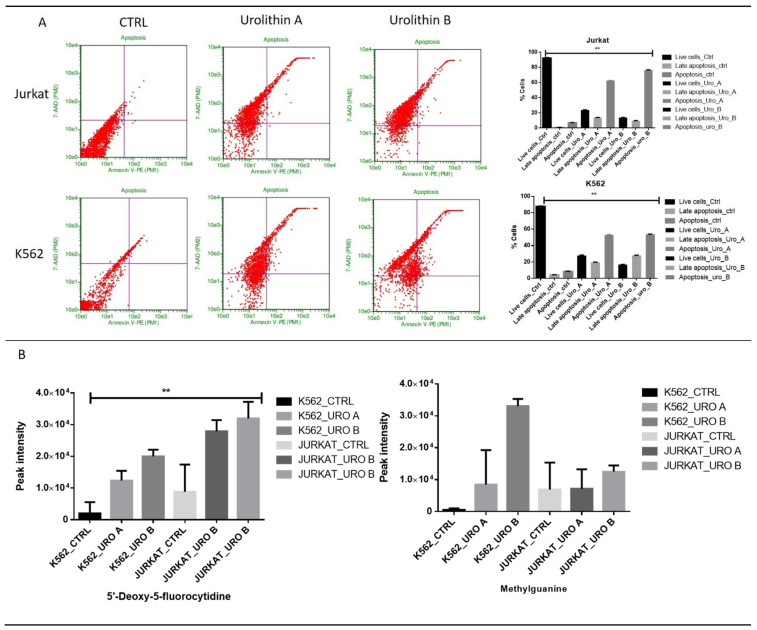
Urolithin A and B induce apoptosis in leukemic cells: (**A**) K562 and Jurkat cells were treated with urolithin A and B; apoptosis assay was performed by using Annexin V assay; (**B**) quantitative levels of various metabolites involved in DNA damage in urolithin A- and urolithin B-treated cells when compared with control, ** *p* < 0.001.

Urolithins have anticancer properties in some cancer cell types. Annexin V-Phycoerythrin and 7-Aminoactinomycin D (7AAD) staining was used to differentiate damaged cells from apoptotic cells. The overall apoptotic rate was calculated as the apoptotic rate of cells in the upper right quadrant (late-stage apoptosis) and the lower right quadrant (early stage apoptosis). We observed that urolithin A and B treatments induce 50% of apoptosis in K562 and 60–70% in Jurkat. Urolithins treatment could inhibit cell proliferation in leukemic cells. We observed that the metabolites associated with oxidative DNA damage methyl-guanine level increased under urolithin treatment (Figure 7A,B).

## 3. Discussion

Urolithin A, B, and C are gut microbiota metabolites obtained from ellagic acid and ellagitannins. Ellagitannins containing products pomegranate, walnut, and berries could be associated with these gut-produced urolithins and their conjugates. Urolithin A and B are crucial metabolites abundant in pomegranate, walnut, and berries. Urolithin A and B have been investigated extensively because of their anticancer effects. However, several reports have shown that UA exerts its anticancer activity on several cancer cell lines [10,17,21,22].

This work provides many insights into the effect of urolithins in leukemic cells and the control of metabolism in cell functions. First, we used untargeted metabolomics to identify novel metabolic and critical pathways affected by urolithin and B treatments. We observed that urolithin A and B treatments involve altering the overall metabolic rate of leukemic cells. The primary critical pathways modulated by urolithins are one-carbon metabolism (cysteine metabolism), lipid metabolism, lactose degradation, and preknown pathways involved in energy metabolism such as the Warburg effect, glycolysis, TCA cycle, which were altered.

We have reported that urolithin treatment in leukemic cells switches cell metabolism toward oxidative phosphorylation (OXPHOS) and increases succinate levels. This metabolic reprogramming plays a significant role in the maintenance of malignant properties. Carnitine is essential to fatty acid oxidation and a potent antioxidant. Carnitines fuel the TCA cycle through β oxidation. Butyrate–carnitine is an anticarcinogenic nutrient that inhibits cancer cell survival. Carnitine has been reported to slow down the development of colon cancer in a murine model [30]. We identified that the high carnitine in urolithin-treated leukemic cancer cells caused the slowdown of cell proliferation. Glutamine metabolism intermediates are essential in endorsing cellular antioxidative defense mechanisms. Glutamate–glutamine contributes to glutathione biosynthesis [23]. The most significant role of glutamine metabolism was to reduce ROS in cancer cells by increasing antioxidants and increasing glutamate dehydrogenase activity to switch energy production from glutamine via the TCA cycle [21]. Our results show that urolithin regulates glutamine uptake, thereby suppressing oxidative stress following leukemic cells.

A signaling network in the cells regulates lipid metabolism. The same lipid molecule can generate different metabolites under different conditions. Some lipids directly induce caspase, which leads to programmed cell death. For example, lysophosphatidylcholine, triglyceride, cholesterol, and lipopolysaccharide [31,32,33] can induce caspase-1 activation. Apoptosis stimulated by fatty acids and their derivatives is associated with activation of multiple caspase-2, -3, -6, -7, -8, and -9 [34,35]. We observed that the urolithin A and B treatments induce fatty acids accumulation in leukemic cells; fatty acids such as prostaglandin, LysoPC, and palmitoleic acid showed increases in their levels in leukemic cells. This fatty acid induction in cellular metabolism is a marker for cells entering apoptosis.

In conclusion, we showed that both urolithin A and B impact cellular metabolism in leukemic cell cancer cells. Urolithins regulate cellular energy metabolic switches necessary for adaptation to oxidative stress and induction of apoptosis. Our results have provided clues for the identification of novel metabolic targets of urolithins in cancer cells.

## 4. Materials and Methods

All Chemicals, biochemicals, kits, and antibodies were of analytical grade and were received from a commercial supplier.

### 4.1. Cell Cultures and Medium Composition

Leukemic cells, namely, Jurkat and K562 cells, were obtained from ATCC, USA. All cell lines were maintained in RPMI (Gibco, Invitrogen, Carlsbad, CA, USA) supplemented with 10% fetal bovine serum (FBS; Gibco one-shot, Brazil), 50 U/mL penicillin, and 50 mg/mL streptomycin (Gibco) at 37 °C and 5% CO2. The cells were treated with either vehicle control or with different concentrations of urolithin A (Sigma SML1791, St. Louis, MO, USA) and urolithin B (Sigma SML1649) for 48 h [34].

### 4.2. Measurement of Cell Proliferation by MTT Assay

Briefly, 1 × 10^4^ cells were seeded in 96-well plates, and cells were treated with different concentrations of urolithin A and urolithin B. After 48 h of treatment, 1:10 ratio 10 µL of MTT (5 mg/mL) were added. Plates were shaken slowly for 5 min. Then, the plate was incubated for another 4 h at 37 °C in a CO_2_ incubator. The media was removed, and 100 µL DMSO was added and incubated for 10 min until all crystals had dissolved, and the OD measured at 540 nm.

### 4.3. Metabolites Extraction

Metabolites were extracted from cells were treated with urolithin A (Uro A) and urolithin B (Uro B). Treated cells were lysed immediately using tissue homogenizer using a combination of ice-cold methanol:acetonitrile:water at a ratio of (2:1:1 *v*/*v*) and vortexed for the 30 s and incubated for 60 min at −20 °C and spin for 15 min at 13,000 rpm at 4 °C. The supernatant was collected, and the samples were taken for LC–MS/MS analysis [35,36,37,38,39].

### 4.4. Mass Spectrometry

Samples were analyzed using an LC–MS/MS LTQ XL™ linear ion trap instrument (Thermo Fisher Scientific, Waltham, MA, USA). Msn parameters, full scanning mode range from 100 to 1000 *m*/*z*. Helium was used as a buffer gas, and nitrogen was used as sheath gas; for run 40, arbitrary units were established as flow rate. The capillary temperature was set at 270 °C and voltage 4.0 V; spray voltage was set at −3.0 kV [34,35,36].

### 4.5. Data Analysis

The raw data were processed using open access to the online XCMS online database. Peaks were searched against human metabolites in the Human Metabolome Database. Pathway analysis and statics were performed using Metaboanalyst [36,37,38].

### 4.6. Apoptosis Assay

The apoptotic cells were detected by using Annexin V-Phycoerythrin and 7-Aminoactinomycin D (7AAD). The cells were treated with Uro A and Uro B. After treatment, cells were washed with PBS three times and resuspended in 100 μL 1X binding buffer and 100 μL Guava Nexin Reagent. After incubation for 20 min in RT (dark condition). The cells were analyzed by flow with the Guava^®^ easyCyte 5 Flow Cytometer, and the percentage of cells that went apoptosis was calculated [36].

### 4.7. Statistical Analysis

Differences between control and urolithin A- and urolithin B-treated groups were determined by one-way analysis of variance (ANOVA) for multiple groups, using GraphPad Prism 8.0 (GraphPad Software, La Jolla, CA, USA). Test results with *p* < 0.05 were considered statistically significant.

## Data Availability

Not applicable.

## Supplementary Materials

The following are available online at https://www.mdpi.com/article/10.3390/ijms22115465/s1.
