# Correction: Alzahrani et al. Urolithin A and B Alter Cellular Metabolism and Induce Metabolites Associated with Apoptosis in Leukemic Cells. *Int. J. Mol. Sci.* 2021, *22*, 5465

**DOI:** 10.3390/ijms27021043

**Published:** 2026-01-21

**Authors:** Abdulaziz Musa Alzahrani, Mohammed Razeeth Shait Mohammed, Raed Ahmed Alghamdi, Abrar Ahmad, Mazin A. Zamzami, Hani Choudhry, Mohammad Imran Khan

**Affiliations:** 1 Biochemistry Department, Faculty of Science, King Abdulaziz University, Jeddah 21589, Saudi Arabia; abdalazizbioc@gmail.com (A.M.A.); razeeth.new@gmail.com (M.R.S.M.); raed.gh1993@gmail.com (R.A.A.); abrar.ahmadg@yahoo.com (A.A.); mzamzami@kau.edu.sa (M.A.Z.); hchoudhry@kau.edu.sa (H.C.); 2 Centre of Artificial Intelligence in Precision Medicines, King Abdulaziz University, Jeddah 21589, Saudi Arabia

There were some errors in the original publication [1], due to the author’s negligence. Some texts need to be updated in Sections 2.7 and 4.6.

In Section 2.7, the sentence “Annexin V-FITC/PI staining was used to differentiate damaged cells from apoptotic cells” should be updated to the following version:

“Annexin V-Phycoerythrin and 7-Aminoactinomycin D (7AAD) staining was used to differentiate damaged cells from apoptotic cells”.

In Section 4.6, the sentence “The apoptotic cells were detected by using Annexin V-FITC and propidium iodide. The cells were treated with Uro A and Uro B. After treatment, cells were washed with PBS three times and resuspended in 100 μL 1X binding buffer and 10 μL Annexin V-FITC and 5 μL PI.” should be updated to the following version:

“The apoptotic cells were detected by using Annexin V-Phycoerythrin and 7-Aminoactinomycin D (7AAD). The cells were treated with Uro A and Uro B. After treatment, cells were washed with PBS three times and resuspended in 100 μL 1X binding buffer and 100 μL Guava Nexin Reagent.”

The authors state that the scientific conclusions are unaffected. This correction was approved by the Academic Editor. The original publication has also been updated.
