# Supplementary figures and images for: Urolithin A and B Alter Cellular Metabolism and Induce Metabolites Associated with Apoptosis in Leukemic Cells
Source: Int J Mol Sci. 2021 May 22;22(11):5465. doi: 10.3390/ijms22115465 (PMC8196872; doi:10.3390/ijms22115465)

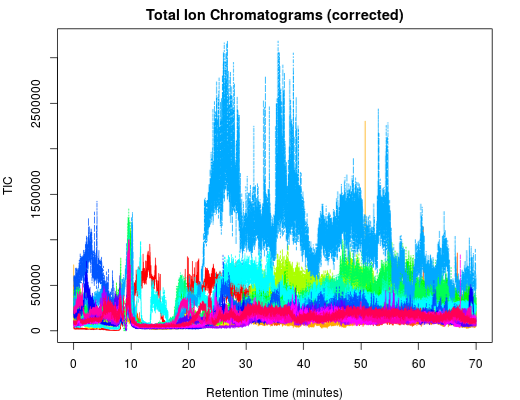

Supplement: Supplementary file 1 [file ijms-22-05465-s001.zip › Supplementry figure 1.tif]
